# Prediction of Post-Concussive Behavioral Changes in a Rodent Model Based on Head Rotational Acceleration Characteristics

**DOI:** 10.1007/s10439-016-1647-x

**Published:** 2016-05-17

**Authors:** Brian D. Stemper, Alok S. Shah, Rachel Chiariello, Christopher M. Olsen, Matthew D. Budde, Aleksandra Glavaski-Joksimovic, Michael McCrea, Shekar N. Kurpad, Frank A. Pintar

**Affiliations:** 1 Department of Neurosurgery, Medical College of Wisconsin, Milwaukee, USA; 2 Clement J. Zablocki Veterans Affairs Medical Center, Milwaukee, USA; 3 Department of Pharmacology and Toxicology, Medical College of Wisconsin, Milwaukee, USA; 4 Neuroscience Research Center, Medical College of Wisconsin, Milwaukee, USA

**Keywords:** Biomechanics, Traumatic brain injury, Angular acceleration, Animal model, Sports, Injury, Concussion, Rotational velocity

## Abstract

Quantifying injury tolerance for concussion is complicated by variability in the type, severity, and time course of post-injury physiological and behavioral changes. The current study outlined acute and chronic changes in behavioral metrics following rotational acceleration-induced concussion in rats. The Medical College of Wisconsin (MCW) rotational injury model independently controlled magnitude and duration of the rotational acceleration pulse. Increasing rotational acceleration magnitude produced longer recovery times, which were used in this study and our prior work as an assessment of acute injury severity. However, longer duration rotational accelerations produced changes in emotionality as measured using the elevated plus maze. Cognitive deficits were for the most part not apparent in the Morris water maze assessment, possibly due to the lower severity of rotational acceleration pulses incorporated in this study. Changes in emotionality evolved between acute and chronic assessments, in some cases increasing in severity and in others reversing polarity. These findings highlight the complexity of quantifying injury tolerance for concussion and demonstrate a need to incorporate rotational acceleration magnitude and duration in proposed injury tolerance metrics. Rotational velocity on its own was not a strong predictor of the magnitude or type of acute behavioral changes following concussion, although its combination with rotational acceleration magnitude using multivariate analysis was the strongest predictor for acute recovery time and some chronic emotional-type behavioral changes.

## Introduction

It is becoming clear that rates of diagnosed concussion in contact sports are increasing. Comparing two studies that reported rates of concussion (per 10,000 exposures) reveals considerable increases for high school sports between the late 1990s and the 2008–2010 seasons.^27^,^38^ The comparison reveals a 94% increase in concussion rate for football, a 162% increase for women’s soccer, and a 144% increase for wrestling. Other studies have highlighted the significant increase in concussion rate in high school sports over a 6-year period, indicating that five of nine investigated sports had statistically significant increases.^32^ While explanations for these increases may include increased awareness about concussions by coaches, medical professionals, and athletes, increased legislation, and better reporting of concussions at the high school level, the possibility of increasing concussion incidence should not be ignored. This comes at a time of increased awareness of the chronic effects of concussions. The significant life-long effects of repetitive mild and moderate brain trauma have garnered considerable media attention. The mechanism behind these neurological declines appears to follow a dose effect phenomenon, as evidenced by boxers with neuropathological burden related to the length of a boxing career and number of bouts.^9^ However, even lower severity or less frequent injuries can lead to chronic difficulties. For example, the work of Guskiewicz, McCrea and colleagues has outlined significantly increased rates of chronic cognitive difficulties and emotional disturbances, including depression, for retired professional football players that had sustained three or more concussions over the course of their career compared to players that had sustained two or fewer.^16^,^17^

The mechanism of concussion has long been associated with head impact resulting in high rate head rotational acceleration.^30^ Head accelerations lead to tensile and shear strains within the brain tissues that can exceed mechanical tolerance resulting in physiologic dysfunction and/or mechanical injury. The science of injury biomechanics would indicate that the onset and outcomes from concussion should then be correlated to head rotational acceleration characteristics, which could include peak magnitude, positive duration, or area under the curve (i.e., rotational velocity). In other areas of injury biomechanics, the risk of soft tissue cervical spine injury has been correlated to peak shear force in the lower cervical spine,^43^ among other mechanical metrics.^3^,^37^ Conversely, the risk of skull fracture is best predicted by the integral of head linear acceleration over time, a metric known as the Head Injury Criterion (HIC).^47^

Biomechanical investigations over the past 40 years have attempted to outline the relationship between head kinematics and concussion. For example, Ommaya and colleagues incorporated data from primate experimentation to highlight the relationship between peak head rotational acceleration and rotational velocity in the onset of concussion.^31^ Margulies and colleagues supported this assertion with results from their porcine model.^28^ More recently, Rowson and colleagues proposed an injury risk curve based on peak head rotational acceleration alone,^35^ and a revised metric based on the combination of head linear and rotational acceleration.^34^ Those injury risk functions were based on head kinematics measured in college football athletes using a helmet-based sensor system. Other combined metrics have also been proposed.^29^ Injury metrics based solely on rotational velocity, such as the BrIC, were also proposed.^44^ That metric compares peak rotational velocities about the three primary axes to computationally derived critical values. A recent assessment of four injury metrics relative to field data from the National Automotive Sampling System (NASS) database demonstrated that HIC and the combined linear/rotational metric from Rowson and colleagues resulted in the highest fidelity, depending on the correlation method.^25^ Both metrics outperformed the BrIC metric. This highlights a considerable lack of consistency between studies and, moreover, a lack of understanding regarding the specific mechanism and tolerance for concussion. Additionally, studies of concussive injury tolerance have been focused primarily on injury onset with very little effort directed toward correlating head kinematics to more chronic outcomes. Given the complexity of concussion, involving primary tissue injury followed by secondary injury associated with pathophysiology of the brain tissues, defining relationships between head kinematics and both acute and chronic outcomes remains important.

Understanding the correlation between head kinematics and the onset or outcomes from concussion is timely, given the increasing incorporation of head impact sensors into the sporting environment. Those sensors have taken the form of helmet-based systems or have been incorporated in mouth guards or patch-based systems that are adhesively attached to the skin.^4^,^5^,^39^,^49^ Most sensors are capable of recording tri-axial linear accelerations and rotational velocities. They transmit that information back to a central hub, which then calculates rotational acceleration, direction of impact, and some injury metrics, including the Head Injury Criterion (HIC). In some cases, the biomechanical information is used to inform clinical personnel on the sidelines about significant head impacts that may be indicative of concussion. However, this is obviously problematic, given the lack of consensus regarding biomechanical metrics associated with the onset of concussion and individual variability for injury tolerance depending on the athlete’s concussive history or genetics. In addition, due to a paucity of experimental and human data, head impact sensors do not account for impact parameters that are likely to influence injury tolerance. For example, injury tolerance is thought to be lower for coronal plane rotational accelerations than rotational accelerations in other directions.^14^,^15^

Therefore, the objective of the current study was to outline relationships between rotational head kinematics and acute and chronic post-injury outcomes, and to identify statistically significant rotational acceleration group-based differences. These relationships were investigated using the Medical College of Wisconsin (MCW) Rotational Injury Model, which produces concussion in rats through pure coronal plane head rotational acceleration. The animal model is ideal for this type of investigation as it eliminates factors associated with changes in injury tolerance including head impact and concussive history and genetics. Additionally, characteristics of head acceleration are strictly controlled and repeatable. Behavioral assessments incorporated in this study are well characterized and focused to reveal changes in specific behaviors.

## Methods

**Figure 1:**
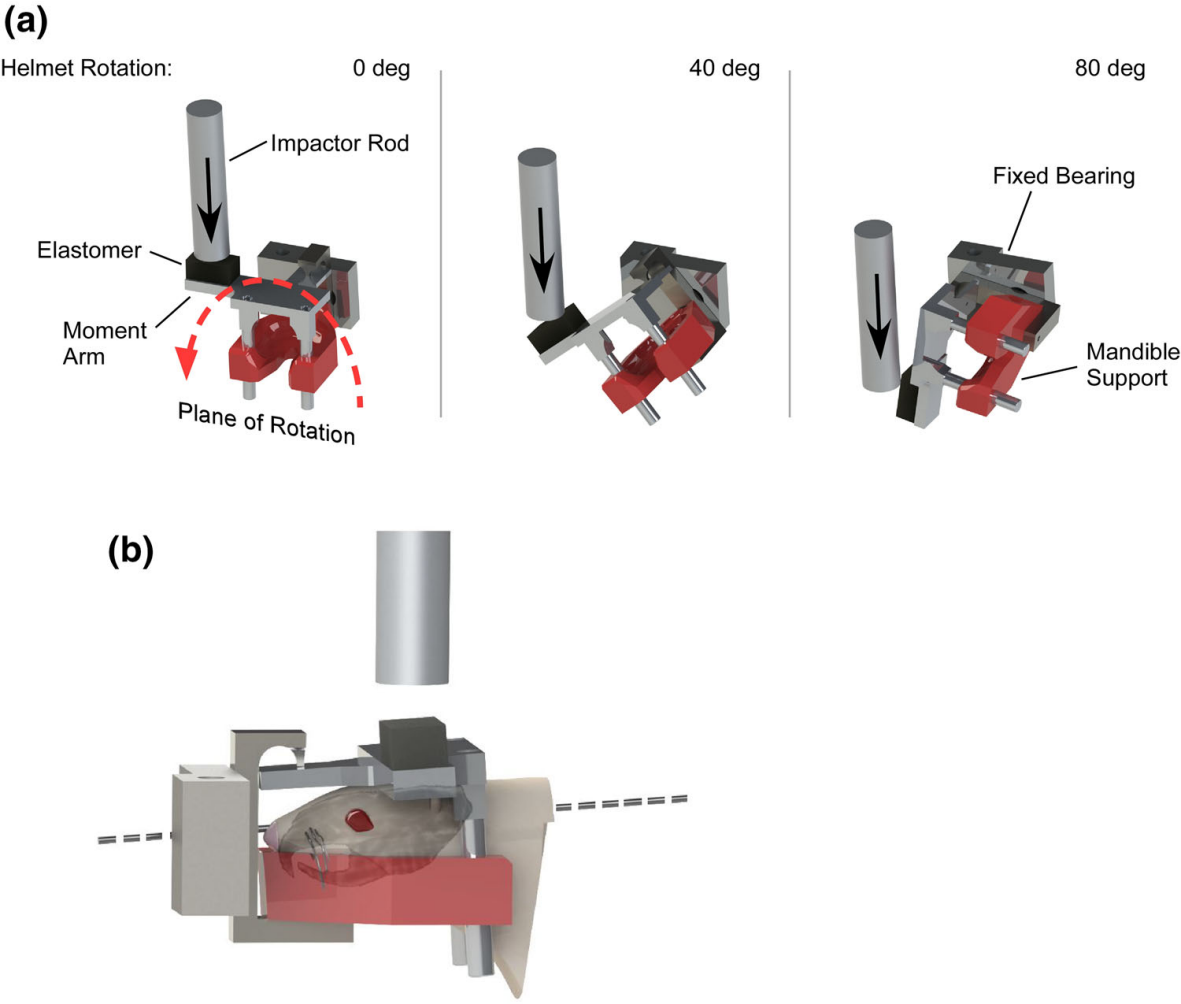
MCW rotational injury device that was used to produce mild TBI in rats through coronal plane rotational acceleration of the head. The three images demonstrate helmet positioning just prior to impact from the rod (left), and during impact causing the helmet to rotate 40° (middle) and 80° (right). Total rotation of the helmet was limited to 90°. Motion was constrained to pure coronal plane rotation, without linear displacement, using a fixed bearing located anterior to the helmet. (b) The axis of helmet rotation was located in the mid-sagittal plane in the lower half of the brain.

All injury and behavioral assessment protocols used for this study were approved by the Institutional Animal Care and Use Committee (IACUC) at our Institution. Concussive injuries were created in rats using the MCW Rotational Injury Model, which consists of a helmet and impactor apparatus (Fig. 1).^42^ The helmet fit securely on the head of the rat without the necessity for surgical intervention, screws, or adhesive,^12^ and was attached to the test frame using a pin joint that allowed pure rotation in the coronal plane of the rat head about the cervical spine. Our prior work using this model resulted in no cervical spine fractures or dislocations, and zero mortality attributable to use of the model.^12^,^42^ Head rotational acceleration was created as the pneumatically-accelerated impactor struck the laterally-extended moment arm of the helmet. This impact resulted in high rate head rotational acceleration through approximately 90° of rotation. Characteristics of the rotational acceleration vs. time pulse were controlled through modulation of the mass and closing velocity of the impactor, and structural characteristics of the elastomer interface.^11^ Those characteristics and rotational acceleration vs. time traces were calibrated prior to rodent injury procedures.

### Injury Protocol

Rats were anesthetized using 4% Isoflurane until unconscious as indicated by a lack of toe pinch reflex. Rats were then given 5.0 mg/kg carprofen *via* injection and placed into the helmet while maintaining anesthesia through a nose cone. The helmet was then aligned and attached to the test frame. Anesthesia was removed and the impactor was immediately launched down the drop tower to strike the moment arm of the helmet and induce helmet rotational acceleration. Following exposure to rotational acceleration, rats were immediately removed from the helmet and placed on a warming blanket for recovery. After return of the righting reflex, rats were returned to their cages, supervised for at least 30 min, and periodically supervised until 6 h post exposure.

### Helmet Kinematics

An accelerometer attached to the distal end of the moment arm measured tangential linear acceleration, which was converted to rotational acceleration of the helmet vs. time. Our previous studies and extensive testing ensured that magnitude and duration of the rotational acceleration vs. time pulse were independently modulated and could be accurately measured for each injury.^42^ Acceleration data were collected at 1.0 MHz (National Instruments Corporation, Austin, TX) and low pass filtered at 10 kHz for analysis. Kinematic measurements of the helmet were used to classify the exposure severity. Peak helmet rotational acceleration was measured as the maximum positive acceleration, and rotational acceleration duration was measured as the positive duration of the acceleration pulse. Rotational velocity was computed as the integral of the positive portion of the rotational acceleration vs. time pulse.

### Injury Groups

A total of five experimental groups were used in the current study, along with a sixth control group that was subjected to the entire experimental protocol including anesthesia and placement in the helmet without exposure to head rotational acceleration. The experimental groups were exposed to different head rotational acceleration vs. time pulses consisting of three rotational acceleration magnitudes of approximately 210, 360, and 500 krad/s^2^, two rotational acceleration durations of approximately 1.5 and 3.5 ms, and four rotational velocities of approximately 140, 250, 380, and 615 rad/s (Table 1).

**Table 1:** Rotational acceleration vs. time characteristics for each of the four experimental injury groups incorporated in this study.

Group	Velocity group	Number	Rotational acceleration (rat)		Rotational velocity (rad/s^2^)	Human equivalent acceleration (rad/s^2^)
			Magnitude (krad/s^2^)	Duration (ms)		
Shams	–	16	–	–	–	–
M1D1	V1	14	215 ± 26	1.8 ± 0.4	140 ± 39	2794
M1D2	V2	38	206 ± 33	3.6 ± 0.4	263 ± 72	2690
M2D1	V2	34	358 ± 46	1.5 ± 0.4	236 ± 78	4652
M2D2	V3	25	368 ± 36	3.4 ± 0.5	383 ± 97	4782
M3D2	V4	19	503 ± 26	3.2 ± 0.2	613 ± 13	6537

M: magnitude of rotational acceleration, D: duration of rotational acceleration. Data are presented as mean ± standard deviation. The total number of rats used for each of the five groups is also included. Rotational acceleration magnitudes measured during rodent experimentation are scaled using Eq. (1) to compute Human Equivalent Accelerations. Due to a lack of any remarkable differences between M1D1 and Shams, the M1D1 group was only used for acute assessments

### Biomechanical Scaling

The magnitude of brain tissue strains for a given rotational acceleration are modulated by brain mass/inertia.^20^ Therefore, the targeted head rotational accelerations incorporated in this study can be scaled using Eq. (1) below.^30^ As such, scaling of targeted rodent head rotational accelerations listed above to the human-equivalent accelerations results in peak head rotational accelerations of 2700, 4650, and 6500 rad/s^2^. Those accelerations would cover the spectrum from mild through classical concussion for the human, according to scaled data from Gennarelli and colleagues.^13^

1 $$\ddot{\theta }_{\text{rat}} = \ddot{\theta }_{\text{man}} \left( {\frac{{M_{\text{man}} }}{{M_{\text{rat}} }}} \right)^{{\frac{2}{3}}}$$

### Acute Severity

Acute injury severity was assessed in this study and our prior work using recovery time. This metric was quantified as the amount of time following removal of anesthesia just prior to injury or sham procedure until return of the righting reflex. Real time videos were obtained during all injury and sham procedures with specific events indicated by the animal technician. Those videos were used to measure the times of anesthesia removal, injury induction, placement on the warming blanket, and return of the righting reflex.

### Behavioral Assessment Protocol

**Figure 2:**
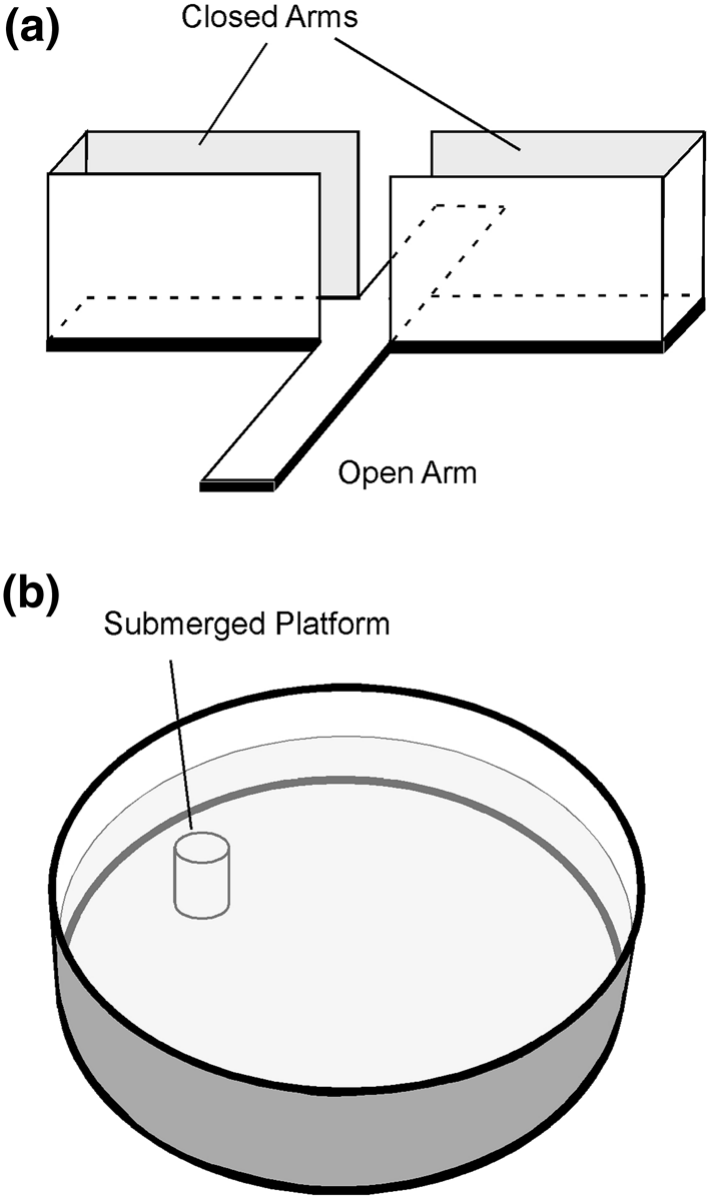
(a) Elevated plus maze (EPM) and (b) Morris water maze (MWM) assessments used to identify post injury changes in activity and emotionality (EPM) and cognitive deficits (MWM).

The elevated plus maze (EPM) assessment (Fig. 2a) was used to quantify activity and emotional-type behaviors following concussion.^7^,^19^ Rats underwent the EPM assessment on day 2 or day 30 post-injury. The maze consisted of four perpendicular 10 × 50 cm arms suspended 82 cm above the floor. A 10 × 10-cm central platform connected the arms. One pair of opposing arms was enclosed by 32-cm-high walls, while the other two arms and the center platform were left open without walls. Rats were initially placed on the central platform facing one of the two open arms. The animals were allowed to explore the maze for 5 min and tracked using a digital video camera mounted above the maze. A computerized tracking system and software (Ethovision V8.0, Noldus Information Technology, Wageningen, The Netherlands) recorded several metrics during each EPM assessment. Those metrics included total distance traveled, total number of arm changes, and the number of entries into and time spent in open areas (center platform and uncovered arms) of the maze. Behaviors associated with post-injury activity included total number of arm changes and total distance traveled. Behaviors associated with changes in emotionality included the number of entries into and the amount of time spent in the open areas of the maze.

The Morris water maze (MWM) (Fig. 2b) Visuo-Spatial Learning Paradigm was used to grade post-traumatic anterograde amnesia and spatial learning following concussion.^8^,^36^ The paradigm consisted of three days of testing on post-injury days 1–3 or 29–31. Separate groups of rats were used for each of the two assessment times because the MWM protocol incorporated in this study relied on the first exposure being a novel environment. Each day of testing included a set of four trials, resulting in 12 trials across 3 days. The four trials per set consisted of initially placing the rats at the four cardinal locations within the 183-cm diameter maze (N, E, S, W), facing the outer wall. During each trial, rats were allowed to swim in the 25-cm deep water until finding and mounting a 10-cm diameter hidden platform submerged 1 cm below the water surface, or until 60 s had passed. The platform was located between the cardinal axes (e.g., SE) halfway between the center and outer wall. Location remained constant for the four trials of a set, but was changed to a novel location in randomized order from set to set. Water temperature remained within one degree of 24 deg C for all trials and the maze was located in a room with numerous visual cues external to the maze and oriented identically for each session. Visual cues were also placed inside the maze. A computerized tracking system and software (Ethovision V8.0, Noldus Information Technology, Wageningen, The Netherlands) recorded several metrics during each MWM trial. Latency to find the hidden platform (sec) was measured for each trial and compared between injury groups and between successive trials/sets. Cognitive deficits were associated as greater latency to find the hidden platform.

### Statistical Analysis

Analysis of variance (ANOVA) was used to determine statistically significant (*p* < 0.05) differences in outcomes between experimental groups. Independent analyses were conducted at acute and chronic time points. Post-hoc analysis was used to conduct pairwise comparisons between individual experimental groups. Linear regression analysis was used to determine characteristics of the rotational acceleration vs. time pulse that best predicted acute and chronic post-concussion outcomes. The following independent metrics were correlated to behavioral outcomes using univariate regression analysis: peak rotational acceleration, rotational acceleration duration, rotational velocity. Likewise, the following combination of metrics were tested using multivariate regression analysis: magnitude and duration of rotational acceleration, and magnitude of rotational acceleration and rotational velocity. Regression analysis was used to determine the ability of those single or combined metrics to predict recovery times, elevated plus maze metrics, and Morris Water maze metrics. Independent regressions were performed for EPM and MWM metrics at acute and chronic time points. Statistical significance of predictors was assessed using *p* values (*p* < 0.05) and the strength of the prediction was assessed using coefficients of determination (*R* ^2^).

## Results

A total of 146 female Sprague–Dawley rats were included in this study, with mean body mass of 289 ± 22 g at the time of the injury or sham procedure. Prior to the injury procedure, rats were exposed to anesthesia for 479 ± 82 s consisting of 5 min in the induction box and nose cone anesthesia for the remaining time until just prior to head rotational acceleration. Group numbers and rotational acceleration characteristics are provided in Table 1.

### Acute Severity

**Figure 3:**
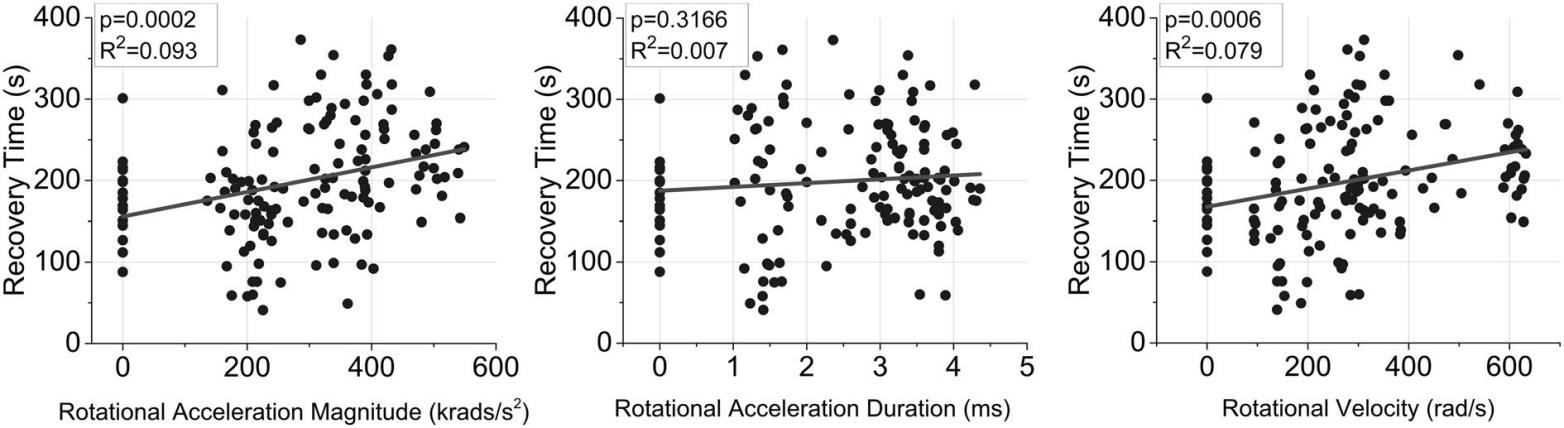
Linear regression relationship between independent rotational acceleration vs. time characteristics and recovery time. Rotational acceleration magnitude and rotational velocity were significant independent predictors for recovery time (*p* < 0.05). The combinations of rotational acceleration magnitude and duration, and rotational acceleration magnitude and rotational velocity were also significant predictors (*p* < 0.05).

Acute severity of injury was assessed using post-injury recovery time. Significant group-based differences were evident for recovery time (*p* < 0.0001) (Table 2). There was no correlation between time under anesthesia and recovery time (*R* ^2^ = 0.00096). Pairwise comparisons demonstrated that all moderate and high magnitude groups (M2, M3) had significantly increased recovery times compared to both the sham group and the low magnitude groups (M1). Linear regression analysis revealed that magnitude of rotational acceleration and rotational velocity were significant predictors for acute recovery time (*p* < 0.001) (Fig. 3). Similarly, the combinations of magnitude and duration, and magnitude and rotational velocity were also significant predictors of acute recovery time (*p* < 0.005) (Table 3). The combination of rotational acceleration magnitude and rotational velocity was the strongest predictor for acute recovery time (*R* ^2^ = 0.098) and magnitude of rotational velocity was the strongest independent predictor (*R* ^2^ = 0.093).

**Table 2:** Acute and chronic post-injury behavioral data for the five experimental injury groups as well as shams.

Metric	*p* value	Sham (0)	M1D1 (1)	M1D2 (2)	M2D1 (3)	M2D2 (4)	M3D2 (5)	Sign. Post Hocs (*p* < 0.05)
*Acute assessments*								
Number		16	14	38	34	25	19	
Recovery time (s)	0.0000	178 ± 52	129 ± 69	181 ± 56	228 ± 86	223 ± 64	222 ± 40	0,1,2–3,4,5
Number		8	14	21	23	16	10	
EPM distance traveled (cm)	0.0024	724 ± 410	954 ± 404	1241 ± 326	1131 ± 392	1263 ± 371	1334 ± 267	0–2,3,4,5 1–2,4,5
EPM arm changes	0.0005	15.6 ± 10.2	22.8 ± 11.1	29.7 ± 8.9	26.8 ± 11.8	34.0 ± 11.8	35.3 ± 8.9	0–2,3,4,5 1–2,4,5 3–4,5
EPM open duration (s)	0.0010	42.2 ± 29.6	58.7 ± 38.2	75.6 ± 39.3	50.7 ± 23.1	78.4 ± 37.1	98.1 ± 29.8	0,3–2,4 0,1,2,3–5
MWM session 1 latency (s)	0.2510	48.8 ± 8.0	37.5 ± 11.3	34.5 ± 11.2	37.8 ± 12.5	38.2 ± 14.1	36.0 ± 11.9	
MWM session 2 latency (s)	0.6503	17.8 ± 10.6	15.8 ± 10.8	23.1 ± 16.4	21.3 ± 18.7	21.5 ± 14.2	15.8 ± 10.7	
MWM session 3 latency (s)	0.6290	21.2 ± 12.4	18.0 ± 15.7	21.2 ± 11.2	18.9 ± 15.0	14.9 ± 7.1	15.2 ± 8.7	
*Chronic assessments*								
Number		8		17	11	9	9	
EPM distance traveled (cm)	0.1012	1317 ± 727		915 ± 331	1168 ± 363	1101 ± 612	777 ± 270	
EPM arm changes	0.0711	29.3 ± 10.7		23.1 ± 11.1	32.1 ± 10.2	24.5 ± 15.0	18.4 ± 8.5	
EPM open duration (s)	0.2132	65.1 ± 35.6		64.9 ± 44.0	90.8 ± 35.3	79.3 ± 48.0	51.9 ± 19.7	
MWM session 1 latency (s)	0.0103	24.1 ± 10.3		41.7 ± 13.1	38.5 ± 13.1	28.0 ± 10.6	31.0 ± 15.4	0–2
MWM session 2 latency (s)	0.3808	18.9 ± 11.9		16.7 ± 8.1	13.0 ± 5.1	20.6 ± 11.1	15.7 ± 8.0	
MWM session 3 latency (s)	0.1465	10.0 ± 3.4		13.6 ± 7.9	10.1 ± 5.5	17.0 ± 10.1	10.5 ± 3.4	

Data are presented as mean ± standard deviation. *p* values represent group-based ANOVA analysis and statistically significant pairwise post hoc comparisons (*p* < 0.05) between groups (0 to 5) are shown. MWM session latencies represent the average latency across the 2nd, 3rd, and 4th MWM trials

**Table 3:** Coefficients of determination (*R* ^2^) for rotational acceleration characteristics as predictors of different post-concussion behavioral metrics.

Metric	Assessment	Magnitude	Duration	Rotational velocity	M + D	M + RV
Recovery time	Acute	**0.093**	0.007	**0.079**	**0.095**	**0.098**
EPM distance traveled	Acute	**0.116**	**0.186**	**0.119**	**0.217**	**0.128**
	Chronic	0.060	**0.113**	**0.136**	**0.125**	**0.138**
EPM arm changes	Acute	**0.139**	**0.209**	**0.180**	**0.249**	**0.181**
	Chronic	0.036	**0.112**	**0.141**	**0.114**	**0.156**
EPM open duration	Acute	**0.069**	**0.158**	**0.097**	**0.170**	**0.097**
	Chronic	0.000	0.015	**0.069**	0.016	**0.014**
MWM latency trial 6	Acute	0.000	0.006	0.000	0.009	0.000
	Chronic	**0.102**	0.067	0.053	**0.120**	0.102

Significant predictors are indicated in bold (*p* < 0.05) or bold and underlined (p < 0.001). M: magnitude, D: duration, RV: rotational velocity

### Emotionality

**Figure 4:**
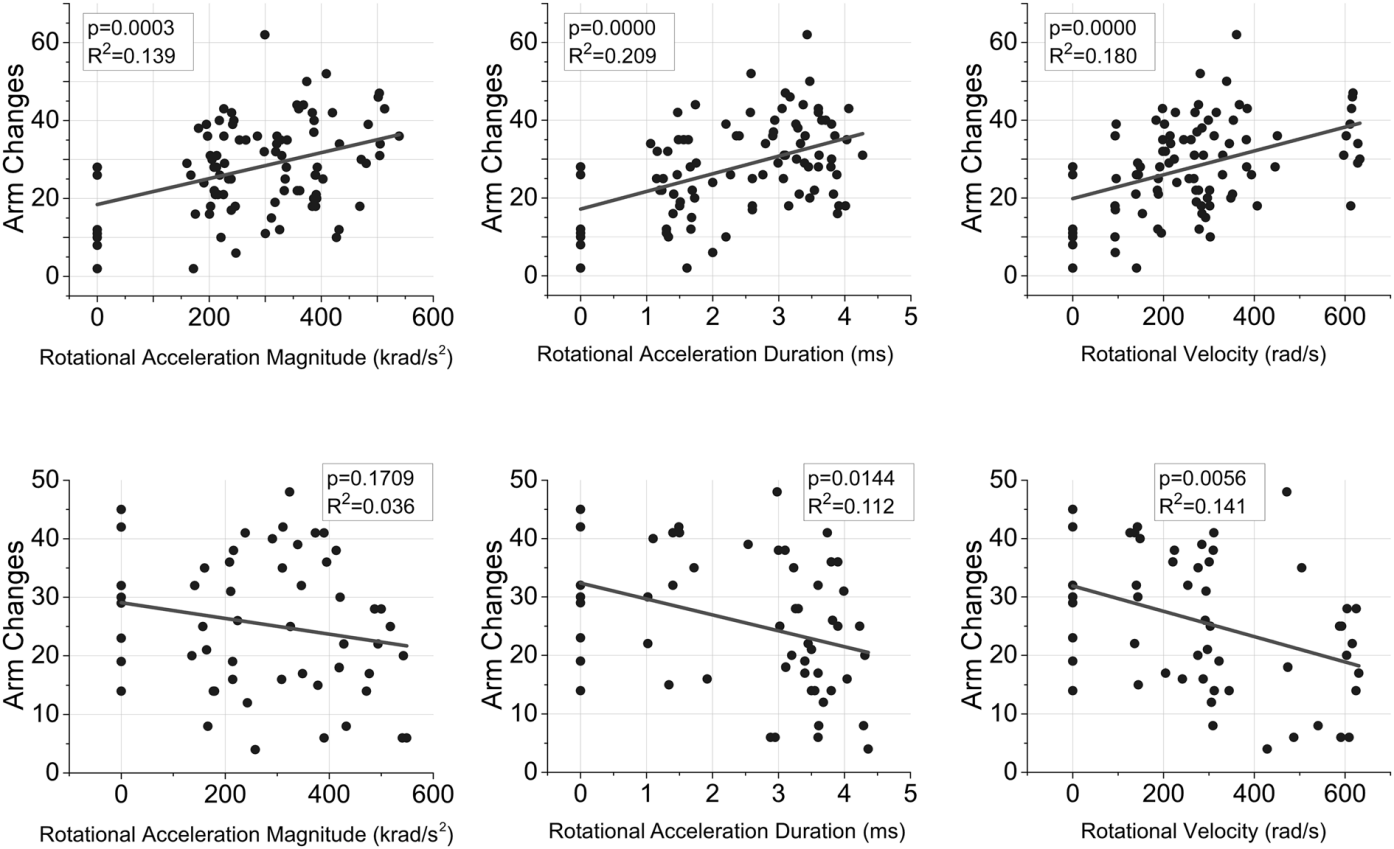
Linear regression relationship between independent rotational acceleration vs. time characteristics and the number of EPM arm changes during acute (upper) and chronic (lower) assessments. Rotational acceleration magnitude and duration, and rotational velocity were significant independent predictors for the number of arm changes at the acute assessment (*p* < 0.05). The combinations of rotational acceleration magnitude and duration, and rotational acceleration magnitude and rotational velocity were also significant predictors at the acute assessment (*p* < 0.05). Rotational acceleration duration and rotational velocity were significant predictors at the chronic assessment (*p* < 0.05). The combinations of rotational acceleration magnitude and duration, and rotational acceleration magnitude and rotational velocity were also significant predictors (*p* < 0.05).

Post injury activity and emotionality were assessed using the elevated plus maze. Assessments were conducted at either acute (2 days post injury) or chronic (30 days post injury) time points. Behaviors at the acute assessment demonstrated significant group-based differences (*p* < 0.05). Specifically, injured rats were more active, assessed as the distance traveled and number of arm changes during the five-minute exposure to the EPM (Table 2). Pairwise comparisons revealed that all injured groups had significantly more acute post-injury activity than controls, with the exception of the M1D1 group (Table 2). Similarly, all long duration groups (D2) had significantly greater activity relative to the M1D1 group. Linear regression analysis demonstrated that all three independent rotational acceleration characteristics (magnitude, duration, rotational velocity), as well as both combinations (magnitude + duration, magnitude + rotational velocity) were significant predictors of acute activity in the EPM (*p* < 0.05) (Fig. 4). The combination of magnitude and duration was the strongest predictor for the number of EPM arm changes at the acute assessment (*R* ^2^ = 0.25 for arm changes) and duration of rotational acceleration was the strongest independent predictor (*R* ^2^ = 0.21 for arm changes) (Table 3).

ANOVA analysis demonstrated no significant group-based trends for EPM activity at the chronic assessment. Duration of rotational acceleration, rotational velocity, and the combinations of rotational acceleration magnitude and duration, and rotational acceleration magnitude and rotational velocity were statistically significantly (*p* < 0.05) correlated to chronic activity in the EPM (Table 3). Rats receiving short duration rotational accelerations had a similar number of arm changes to shams, demonstrating progressively fewer arm changes for the M1D2 and M2D2 groups, followed by the M3D2 group. In other words, the highest velocity group (M3D2, V4) had the fewest arm changes (Table 2). Distance traveled in the EPM demonstrated a somewhat similar trend.

**Figure 5:**
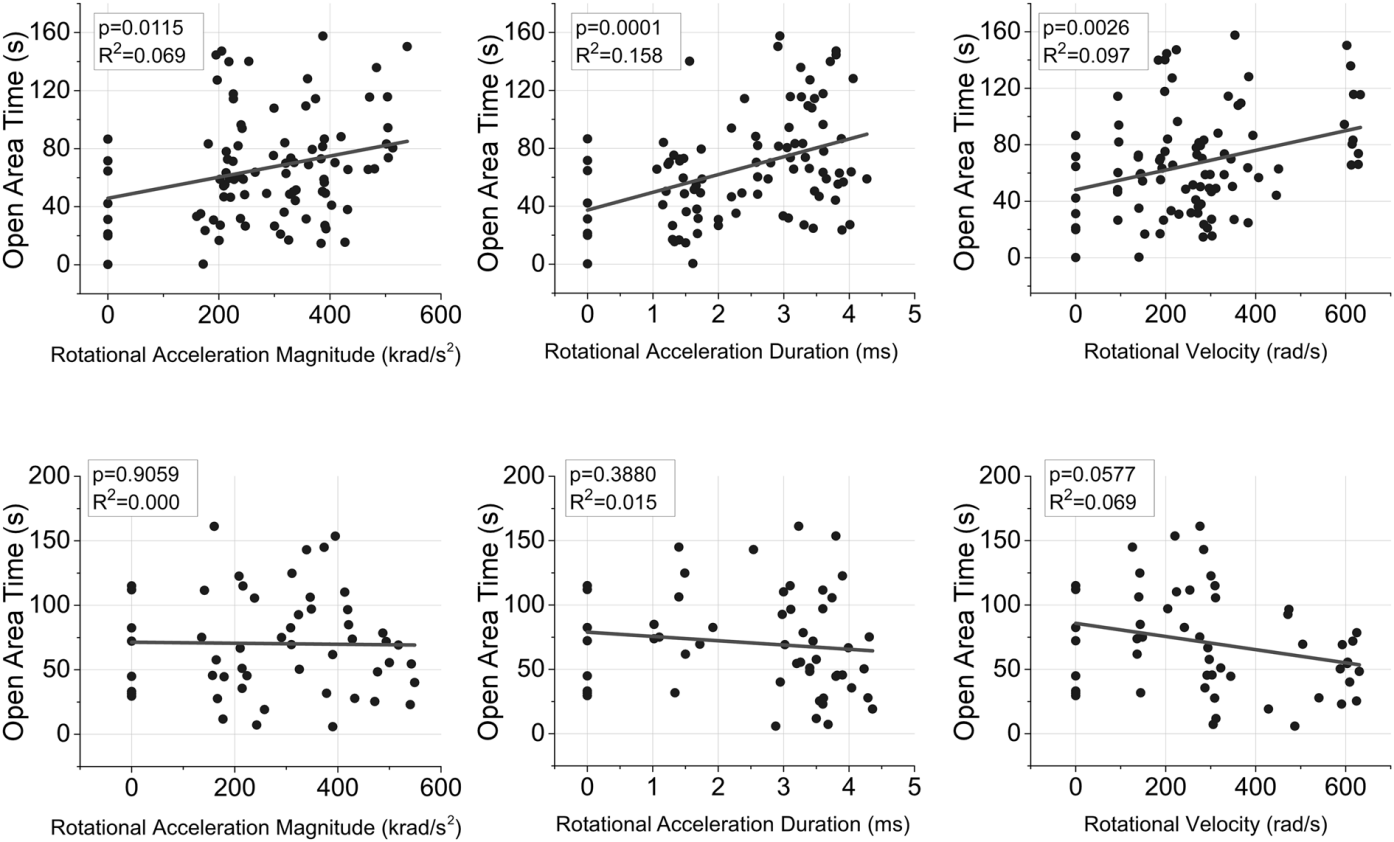
Linear regression relationship between independent rotational acceleration vs. time characteristics and the duration of time spent in the open areas of the EPM during acute (upper) and chronic (lower) assessments. Rotational acceleration magnitude and duration, and rotational velocity were significant independent predictors for open area duration at the acute assessment (*p* < 0.05). The combinations of rotational acceleration magnitude and duration, and rotational acceleration magnitude and rotational velocity were also significant predictors at the acute assessment (*p* < 0.05). In the chronic phase, rotational velocity approached statistical significance (*p* = 0.058) and the combination of rotational acceleration magnitude and rotational velocity was a significant predictor a of open area time (*p* < 0.05).

Time spent in the open areas of the elevated plus maze during the acute assessment demonstrated significant group-based differences (*p* = 0.001). In general all groups receiving long duration rotational accelerations (D2) spent more time in the open areas than the shams and short duration groups (D1), which was supported by statistically significant post hoc pairwise comparisons (Table 2). Linear regression analysis demonstrated that all three independent rotational acceleration characteristics (magnitude, duration, rotational velocity), as well as both combinations (magnitude + duration, magnitude + rotational velocity) were significant correlates with time spent in the open areas of the EPM during the acute assessment (*p* < 0.05) (Fig. 5). The combination of magnitude and duration was the strongest predictor (*R* ^2^ = 0.17) and duration of rotational acceleration was the strongest independent predictor (*R* ^2^ = 0.158).

Group-wise trends for open area time disappeared by the chronic assessment time point, although injured rats generally spent progressively less time in the open areas of the EPM with increasing rotational velocity (Table 2). However, linear regression analysis indicated that only the magnitude of rotational velocity and the combination of rotational acceleration magnitude and rotational velocity were significant correlates (*p* < 0.05) with open area time at the chronic assessment (Fig. 5).

### Cognitive Deficits

**Figure 6:**
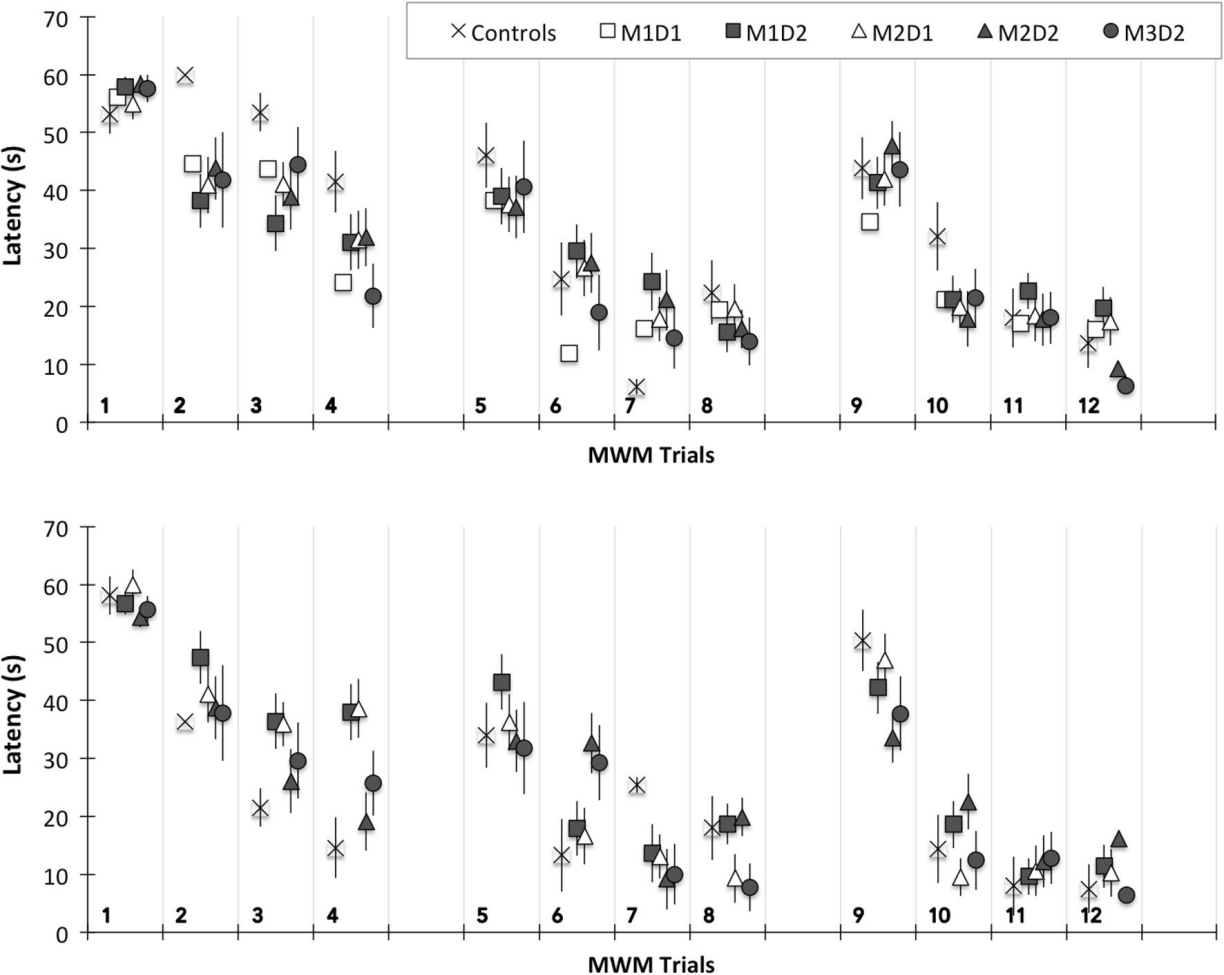
Latency to find the hidden platform in the Morris water maze assessment for each of the four trials per set and all three sets of the assessment. Data are presented as mean and standard error for each of the six groups incorporated in this study. Data for the acute assessment are presented in the upper figure and data for the chronic assessment are presented in the lower figure.

Cognitive deficits were assessed using the Morris Water maze. Assessments were conducted at acute (days 1–3 post injury) or chronic (days 29–31 post injury) time points. Although average latency to find the platform during the first session at the chronic assessment demonstrated significant group-based differences, group-based trends were not biomechanically relevant. No other significant group-based cognitive deficits were identified. However, latency to find the platform was significantly dependent on trial number (*p* < 0.05). For all groups, latency decreased from the first to the fourth trial of each set and the slope of decreasing latency became steeper from the first through the third sets (Fig. 6). These trends indicate that all groups were experiencing a spatial learning process. Linear regression revealed that latency to find the hidden platform demonstrated statistically significant correlation (*p* < 0.05) during only trial 6 at the chronic assessment. Latency to find the hidden platform was significantly correlated to magnitude of rotational acceleration (*R* ^2^ = 0.102) and the combination of magnitude and duration of rotational acceleration (*R* ^2^ = 0.120).

## Discussion

A primary objective of this study was to identify significant correlations between head rotational acceleration characteristics and acute and chronic behavioral outcomes following concussion. In general, magnitude and duration of head rotational acceleration correlated best with acute post-injury behaviors, whereas chronic behaviors correlated poorly with injury biomechanics, although statistically significant correlations (*p* < 0.05) were evident between rotational acceleration duration and rotational velocity, and some EPM metrics during the chronic assessment. The combination of rotational acceleration magnitude and duration was consistently the best predictor for acute post-concussion behaviors. However, that combination was often dominated by either magnitude or duration. For example, acute post-injury activity assessed using distance traveled in the EPM was best correlated to the combination of magnitude and duration (*R* ^2^ = 0.217). However, the correlation between distance traveled and duration of rotational acceleration was only marginally lower (*R* ^2^ = 0.186), indicating that duration alone had almost the same effect on acute post-injury activity as the combination of magnitude and duration. This trend was consistent across all correlations of injury biomechanics with acute post-injury behaviors, wherein EPM metrics associated with acute activity and emotionality were most strongly correlated to duration of rotational acceleration and acute recovery time was most closely correlated to the magnitude of rotational acceleration. Coefficients of determination for the other independent metrics, rotational acceleration duration and rotational velocity in the case of recovery time and rotational acceleration magnitude and rotational velocity in the case of EPM metrics, were much lower (Table 3). This indicates that those metrics were less associated with the specific behaviors outlined in those assessments and highlights a complex relationship between head rotational acceleration characteristics and the type and time course of post-injury outcomes. These findings demonstrate independent roles for rotational acceleration magnitude and duration in the prediction of acute outcomes following concussion, and a possible role for rotational velocity in chronic outcomes. These differences are likely attributable to differing stress and strain profiles (i.e., magnitude, distribution, duration) that will affect and injure different regions of the brain with varying severity.^26^ The external manifestation of this phenomenon is differing behavioral outcomes and variation in the time course of these changes, as demonstrated in this study.

Present results demonstrated that magnitude of rotational acceleration was strongly correlated with acute severity of injury. This finding is consistent with much of the prior work focusing on concussion onset that either indicated peak magnitude of head rotational acceleration or a combination of peak magnitude and rotational velocity were responsible for the onset and acute severity of concussion. For example, Rowson and colleagues reported an injury risk curve, derived using head impact sensor data from college football athletes, describing increased concussive injury risk with increasing magnitude of head rotational acceleration.^35^ Additionally, Gennarelli and colleagues incorporated scaled experimental data from primates to demonstrate increasing concussive injury severity with greater head rotational acceleration magnitudes.^14^ That study identified a linear relationship between graded levels of injury severity from mild concussion through severe diffuse axonal injury with increasing rotational acceleration magnitudes between 3.0 and 16.5 krad/s^2^. Similarly, using a porcine rotational acceleration model, Ibrahim and colleagues demonstrated more severe injuries for higher magnitude rotational accelerations that included marked EEG amplitude suppression and significantly more severe brain hemorrhage, ischemia, and axonal injury.^21^ Computational modeling incorporating detailed finite element models has explored the issue on a tissue level. Takhounts and colleagues indicated that a cumulative strain damage measure (CSDM) correlated well with rotational acceleration magnitude as well as rotational velocity.^45^ Results of that study demonstrated increasing CSDM for greater rotational acceleration magnitudes. Greater CSDM values were associated with a greater risk of diffuse axonal injury. CSDM magnitude, however, was more strongly correlated with head rotational velocity. Nonetheless, present findings that acute injury severity was best predicted by peak head rotational acceleration is supported by findings from the prior work cited above that indicated greater injury risk and greater injury severity with increasing head rotational acceleration magnitude.

This study also demonstrated increased activity and open area time during the acute assessment for injured rats in the elevated plus maze. Although all three head kinematics metrics were significant correlates of EPM behavior in the acute phase, rotational acceleration duration was the strongest significant independent predictor according to the coefficient of determination. Likewise pairwise group comparisons demonstrated that only groups receiving longer duration rotational accelerations demonstrated significantly increased activity relative to shams. The finding of greater post-injury open area time is somewhat contradictory to prior studies that reported greater closed arm and less open arm time following fluid percussion or blast injuries. For example, Jones and colleagues reported increased anxiety-like behavior characterized by increased EPM closed arm time at multiple time points following severe fluid percussion injury.^23^ Similarly, Awwaad and colleagues reported transient anxiety-like symptoms at day 9 post-injury that were associated with less open arm time following blast injury exposure.^2^ Those changes resolved by more chronic post-injury time points. Contradictory findings between the current and prior studies incorporating different injury models is likely attributable to the mechanism of injury.^41^ Other studies using the rotational acceleration injury mechanism demonstrated similar EPM behaviors at acute time points. For example, following sagittal plane rotational acceleration (1.5 Mrad/s^2^), rats demonstrated non-significant increases in the EPM open arm time (+30%) on the third day post-injury.^33^ This clearly demonstrates the importance of injury mechanism on outcomes following concussion and underlines the importance of the present findings that demonstrated the significant effects of head impact biomechanics on the type, severity, and time course of specific outcomes.

Another major outcome from this study was a lack of significant group-based evidence of cognitive deficits at acute or chronic time points. This finding is generally consistent with our prior work using the rotational model,^42^ although we previously demonstrated statistically significant cognitive deficits using our shockwave injury model.^6^ However, the current study was able to identify significant correlations between latency to find the hidden platform during MWM trial 6 at the chronic assessment and magnitude of rotational acceleration. Latencies increased with rotational acceleration magnitude. Although not evident during other MWM trials, increased latency during the second MWM set may indicate late-developing cognitive deficits associated with decreased cognitive flexibility. As indicated above, the protocol consisted of changing the platform location between MWM sets, with four trials per set. Longer latencies following platform relocation would be indicative of an inability to adjust to the new platform location, an effect identified in other investigations involving sleep apnea and drug abuse.^24^,^46^ Additionally, although our group has not previously identified late-developing cognitive deficits following rotational acceleration injury, our prior blast TBI studies in rodents identified cognitive changes during chronic assessments that were either not present at the acute assessment^41^ or persisted and advanced from those identified at the acute assessment.^6^ These cognitive changes, identified in the current rodent model, may be associated with post-concussive syndrome in the human.

A possible explanation for the lack of consistent cognitive deficits in the current study could be the focus on lower severity injuries. When scaled from the rat to the human using a brain mass scaling technique described earlier, peak head rotational accelerations for the three groups were approximately 2700, 4650, and 6500 rad/s^2^. The two lower magnitudes would be associated with much less than 10% injury risk according to data derived from humans.^35^ The highest magnitude scaled acceleration would be associated with approximately 60% injury risk according to that data. This highlights another major outcome from this study, in that significant and lasting changes in emotionality can result from head impacts that do not produce significant cognitive deficits. This may point to either a lower biomechanical threshold for changes in emotionality or again highlight the importance of head rotational acceleration vs. time characteristics in the modulation of different outcomes following concussion.

Application of these animal model-based findings to the human environment is clear. Whereas a majority of contemporary experimental animal-based research is conducted using injury models not focused on accurate biomechanics, our group, along with a limited number of other research groups,^10^,^18^,^28^,^50^ has focused on the development of experimental injury models that match the physics of human concussion and can be used to understand the mechanism and tolerance of concussion in the human as well as biomechanical factors that influence injury severity and outcomes. As such, a critical aspect of the human relevance of this study is that some of the variability in patient outcomes can and should be attributed to the biomechanics of the event. For example, based on the data presented here, one would expect that concussion from a long duration head impact, such as motor vehicle airbag loading, would lead to a different post-injury behavioral phenotype than a short duration head impact that may be experienced in blunt trauma assaults. Recent studies of clinical outcomes following concussion have highlighted this variability, reporting 26 to 31% of patients with anxiety and other mood disorders beyond post-traumatic stress disorder (PTSD).^48^ Results of the study would imply that some of that variability can be attributed to details of the concussion event. While this makes sense from a biomechanical perspective, to our knowledge, this remains the first study to highlight the differing effects of rotational acceleration magnitude and duration, and rotational velocity on the type and time course of behavioral changes following concussion. Likewise, this information has not been incorporated into the clinical environment although considerable ongoing research is required before this goal is realized. Nonetheless, the findings outlined in this study can lay the foundation for future animal- and human-based research focusing on further defining these relationships.

Therefore, the current research is timely, given the increasing incorporation of head impact sensors in military and sporting environments. Laboratory-based experimental findings from biofidelic models can be used to shape human study hypotheses on injury mechanisms and focus or explain ongoing analyses. However, the converse situation is also true in that animal models can be used to better understand mechanisms or trends observed in human studies. For example, our preliminary data from an ongoing study identified a correlation between decreasing head rotational accelerations at the time of concussion with increasing number of significant prior subconcussive head impacts.^40^ However, that study included a limited number of concussed athletes (*n* = 19). Therefore, animal experimentation incorporating a biofidelic injury model can be used to better understand mechanisms of possible progressive tissue damage associated with repetitive subconcussive head impacts that may decrease injury tolerance and increase individual susceptibility. Given that the scope of human studies of concussion are often somewhat limited, incorporation of biofidelic animal models may provide a useful supplement to better understand trends and shape analyses.

Perhaps the most obvious limitation of this study is the limited strength of correlations between injury biomechanics and behavioral outcomes. The strongest correlations presented here had coefficients of determination between 0.2 and 0.25. A primary reason for the relatively weak correlations, of course, is variability in rodent behavioral response. Coefficient of variation (standard deviation divided by mean) was between 25 and 70% for the primary EPM metrics, including arm changes and open area durations. However, high variability is a limitation common to most *in vivo* studies, particularly in the area of animal behavior. The coefficients of variation for similar EPM metrics from other concussion studies were consistent with the current findings (35 to 68% for open area duration),^22^,^33^ and in some cases exceeded 100%.^1^ Therefore, somewhat weak correlations were expected due to the inherent variability of rodent behavioral data. However, as outlined in Table 3, only statistically significant correlations were discussed in this manuscript and used to form the conclusions of the study. Additionally, statistically significant group-based trends supported the correlation data, as shown in Table 2, which provides added confidence to the conclusions of this study.

## Conclusions

This study incorporated a unique experimental rotational acceleration injury model for concussion to demonstrate for the first time that characteristics of the head rotational acceleration vs. time pulse influence the type and time course of post-injury behavioral changes. During the acute phase, magnitude of head rotational acceleration was most strongly correlated to recovery time, our assessment of injury severity, and duration of head rotational acceleration was most strongly correlated to changes in emotionality that manifested as increased activity and open arm duration in the elevated plus maze. In the chronic phase, rotational velocity had a weak, although statistically significant, correlation to changes in emotionality. Statistically significant group-wise trends supported these correlations. These findings have significant implication for understanding concussive injury mechanisms and the continued development of injury tolerance metrics. Coupled with human studies, these findings lay the foundation for a better understanding of the complex clinical situation associated with concussion and post concussion syndrome.
